# How to Improve Entrepreneurship Education in “Double High-Level Plan” Higher Vocational Colleges in China

**DOI:** 10.3389/fpsyg.2021.743997

**Published:** 2021-10-13

**Authors:** Yangjie Huang, Yanzi Zhang, Zehai Long, Da Xu, Ruijie Zhu

**Affiliations:** ^1^School of Education, Hangzhou Normal University, Hangzhou, China; ^2^Department of Student Affairs, Zhejiang Industry & Trade Vocational College, Wenzhou, China; ^3^Institute of China Innovation and Entrepreneurship Education, Wenzhou Medical University, Wenzhou, China; ^4^College of Landscape Architecture, Zhejiang A&F University, Hangzhou, China

**Keywords:** “Double High-level Plan”, entrepreneurship education, entrepreneurship education performance, vocational education, empirical study

## Abstract

Entrepreneurship education complements vocational education in helping students develop their career prospects. This empirical study comprehensively analyzed sample data of 13,885 students from 40 “Double High-level Plan” higher vocational colleges in China using robust standard error regression analysis and other methods. The results showed that Entrepreneurship Practice (EP), Entrepreneurship Curriculum (EC), and Integration of Entrepreneurship Education and Professional Education (IEEPE) have a significant positive effect on Entrepreneurship Education Performance (EEP), with EP being the most important factor. Furthermore, ascribed factors (gender, household registration, only child or not, whether parents have entrepreneurial experience) and self-achieved factors (double high-level type, school area, subject major, whether to accept social entrepreneurship education) were found to affect students' perception of investment in entrepreneurship education. The study summarizes the existing problems of entrepreneurship education in “Double High-level Plan” higher vocational colleges and proposes four suggestions: pursue the integrated development of entrepreneurship education and “Double High-level” construction, advance both theoretical education and practical education, promote digital reform of the “three teaches” (teachers, teaching materials, and teaching methods), and develop entrepreneurship education in a comprehensive and balanced manner. This has certain theoretical and practical significance for the improvement of entrepreneurship education in other developing countries.

## Introduction

New scientific and technological developments are promoting the digitalization of global industries and digital industrialization, and there is an urgent need to train a large number of specialized talents with digital literacy and skills. As an education type closely related to industry, vocational education responds to real-world needs. The Education 2030 Framework for Action (FFA) has been published by UNESCO to promote lifelong learning for all and global sustainable development (UNESCO, 2015). The United States has introduced Strengthening Career and Technical Education for the twenty-first Century Act to strengthen investment in and satisfaction with vocational education (CBO, 2018). Germany has launched the framework initiative of “Berufsbildung 4.0” to cultivate digital technology capabilities for Industry 4.0 (BMBF, 2017). Although there are differences in higher vocational education around the world, almost all countries face difficulties such as slow response to labor market demands, difficulty in balancing the needs of students and enterprises, insufficient integration of industry and education, and weak faculty (OECD, 2010). Moreover, the policy push to promote social equality is allowing more disadvantaged students to receive higher education; this has led to a decreased quality reduction and a corresponding neglect of vocational education. The latter is not only overshadowed but also often treated as “inferior” to academic education (Bathmaker et al., 2018). Entrepreneurship education (EE) is an important breakthrough to promote the comprehensive reform of higher vocational education. It can promote innovation of teaching concepts and paradigms; foster the close integration of stakeholders such as government, industry, education, and research; and continuously integrate discrete and cross-border knowledge. This would help achieve a rapid iteration of new knowledge, new theories, and new technologies (Wilson, 2008; Wang and Tian, 2018) so as to cultivate innovative talents with advanced technical skills to adapt to the economic and social development; it would also promote the development of higher vocational education.

As China's economy develops rapidly, its industries are faced with huge challenges in structural optimization, transformation and upgrading. Although China has the world's largest population and labor force, and has entered the stage of universalizing higher education, there is still a severe shortage of skilled workers. Data show that by 2017, the total number of skilled workers in China is 165 million, among which 47.91 million are highly skilled, accounting for 29.03%, which is far behind countries such as Germany and the United States (more than 40%) (Li and Xing, 2020). In 2019, China released the National Plan for The Implementation of Vocational Education Reform, putting vocational education on an equal footing with general education. Subsequently, policies were introduced to build high-level vocational colleges and majors with Chinese characteristics to improve the quality and usefulness of vocational education. In 2020 and 2021, China over fulfilled its goal of expanding the number of higher vocational students by 2 million. The reform of China's vocational education is unprecedented, and the scale of higher vocational students is huge. How to improve the quality of vocational education by developing EE to promote the employment of graduates and meet the needs of economic and social development is of great significance to the international community, especially developing countries.

## Literature Review

### Entrepreneurship Education

EE in China started with the “Challenge Cup” National College Student Business Plan Competition held by Tsinghua University in 1997. Since 2015, Chinese governments at all levels have issued policies to rapidly expand EE. EE is not an education for the “minority,” it provides “broad-spectrum” training to foster innovative consciousness, innovative thinking, innovative spirit, and entrepreneurial ability (OECD, 2009). It can change learners' entrepreneurial attitude and enhance their entrepreneurial willingness (Caggiano et al., 2016; Handayati et al., 2020), thus promoting their choice of entrepreneurship (Shabbir et al., 2016) and having a positive effect on entrepreneurial performance (Ho et al., 2018). The United States was the first country to provide EE. Although it is still considered not completely “legal” (Kuratko, 2005; Jones and Matlay, 2011), it has gradually formed a well-rounded EE system integrating curriculum teaching, practical teaching, and disciplinary and professional education (Wilson, 2008; Xia and Mao, 2020). The study draws on Huang and Huang (2019) evaluation of the process dimension of EE and divides EE into three dimensions: Entrepreneurship Curriculum (EC), Entrepreneurship Practice (EP), and Integration of Entrepreneurship Education and Professional Education (IEEPE).

EC. In 1947, professor Myles Mace of Harvard University opened the first EC, “Management of New Enterprises.” Nowadays, more than 3,000 universities around the globle offer ECs (Turner and Gianiodis, 2017), and three types are divided by scholars: about entrepreneurship, for entrepreneurship, and through entrepreneurship (Robinson et al., 2016). ECs teach theoretical knowledge, provide a toolkit for starting a business, and allows students to experience entrepreneurial activities. These are conducive to popularizing entrepreneurial knowledge, promoting entrepreneurial ideas, stimulating entrepreneurial awareness, and playing a very important “inspiring” role (Piperopoulos and Dimov, 2015).

EP. EP is widely advocated because EE need to connect to the real environment (Edelman et al., 2008). Entrepreneurs need a buffer period to gain practical experience known as “tacit knowledge” (Hellmann and Thiele, 2019; Buttler and Sierminska, 2020), and EP can help them gain skills, knowledge and mindset, and enhance entrepreneurial capacity and performance through action-based activities (Radianto and Santoso, 2017; Neck and Corbett, 2018).

IEEPE. IEEPE is the third wave of EE development in the US (Yin et al., 2021). The institutionalization of EE has intensified the diffusion of EC and EP outside traditional business school settings, for example, they have been integrated into programs of STEM, medical science and humanities and arts (Turner and Gianiodis, 2017). IEEPE will enable more students to receive EE and promote more professional-based and innovation-based entrepreneurship.

### Entrepreneurship Education Performance (EEP)

In 1966, Stufflebeam, a famous American evaluation expert, presented the CIPP education evaluation model, which includes four aspects: context, input, process, and product. From the perspective of outcome dimension, the evaluation of EE focuses more on entrepreneurial intention, entrepreneurial potential, entrepreneurial rate, entrepreneurial number, entrepreneurial situation, etc. (Neck and Corbett, 2018; Xu, 2019). However, the establishment of enterprises is not the only goal of EE, and the selection of the above indicators cannot reflect the EEP for most people. In this regard, Xu (2019), based on Chinese practice, proposed the three-dimensional three-level structural VPR evaluation model and Huang and Huang (2019) built a full-chain evaluation system, both aiming at promoting comprehensive and scientific evaluation. This study focuses on EEP from the perspective of “the public” rather than “the minority,” that is, it looks at the changes in students' entrepreneurial knowledge, innovative spirit, entrepreneurial skills, and entrepreneurial willingness, as well as their satisfaction with EE.

### Hypotheses

**Figure 1:**
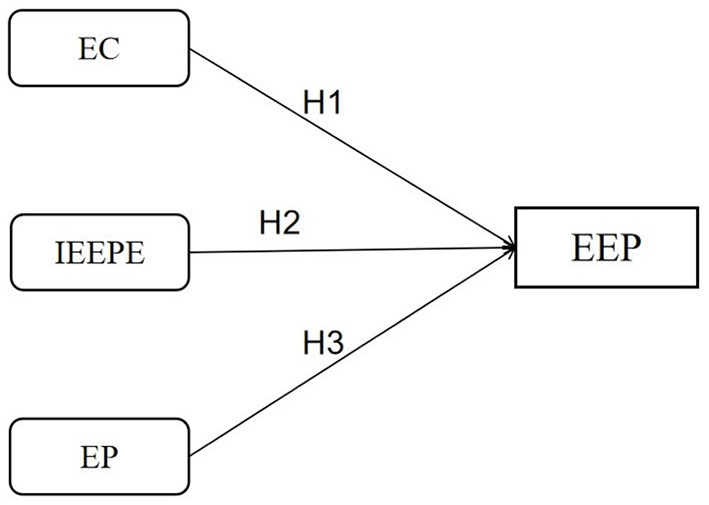
Research framework of EEP.

After years of exploration and practice, EE in higher vocational colleges has achieved rapid growth. However, ECs are separated from professional courses, and replace curriculum teaching with practical activities, that EE is still in the primary stage and has not played a better role. With the “plan of construction of high-level vocational schools and majors with Chinese characteristics” (“Double High-level Plan”) being implemented in more than 1,400 higher vocational colleges, how do students evaluate EE? What factors affect the EEP? How to promote better, faster, and higher quality development of EE? According to the above literature review, this paper presents three hypotheses, and Figure 1 shows the hypothesized structural model.

H1: EC has a positive effect on EEP.
H2: IEEPE has a positive effect on EEP.
H3: EP has a positive effect on EEP.

## Materials and Methods

### Data Collection

The data were collected through a survey of students and graduates who had received EE in 1,231 colleges and universities across the country from 15 September 2018 to 18 January 2019. People who have received EE can better evaluate EE, so the survey didn't include first-year college students who had recently enrolled. We used a paid questionnaire tool called “Questionnaire Star” to collect and summarize the data, and *via* IP restrictions to limit each person to one answer. In total, 170,764 valid questionnaires (valid ratio of 90.87%) were obtained after excluding invalid questionnaires such as invalid school name and short answer time. The primary principle of data selection is the pertinence of the research object—“Double High-level Plan” higher vocational colleges which have a good quality of vocational education in China. Based on the list (197 colleges) published by the Ministry of Education and the Ministry of Finance in December 2019, 83 colleges (14,075 samples) were selected. And finally, 40 colleges (13,885 samples) with a minimum sample size of 30 were selected due to the sample size <30 is small and unrepresentative. The samples covered 18 provinces and municipalities in China, including 10 in the east, 4 in the central and 4 in the west. The basic information is shown in Table 1.

**Table 1:** Basic information of students (*N* = 13,885).

**Items**		**Frequency**	**Percentage** **(%)**
Gender	Male	7,008	50.47
	Female	6,877	49.53
Only child or not	Yes	5,188	37.36
	No	8,697	62.64
Double high-level type	High-level School Construction College (Grade A)	1,527	11.00
	High-level School Construction College (Grade B)	1,076	7.75
	High-level School Construction College (Grade C)	1,273	9.17
	High-level Major Group Construction College (Grade A)	616	4.43
	High-level Major Group Construction College (Grade B)	5,669	40.83
	High-level Major Group Construction College (Grade C)	3,724	26.82
School area	Eastern China	11,409	82.17
	Central China	1,445	10.41
	Western China	1,031	7.42
Subject major	Science and Engineering	8,107	58.40
	Economics and Management	4,547	32.70
	Other	1,231	8.90
Entrepreneurial practice experience	Yes	2,769	19.94
	No	11,116	80.06
Entrepreneurial choice	Yes	2,616	18.84
	No	11,269	81.16
Whether parents	Yes	3,582	25.80
have entrepreneurial experience	No	10,303	74.20
Household	Urban	3,534	25.45
registration before college entrance examination	Rural	1,0351	74.55

The questionnaire comprised 31 questions, included students' basic information (gender, grade, etc.), the current state of EE at their respective schools, and an evaluation of their satisfaction. The types of questions included single chioce, multiple-choice ranking, and scale questions. A five-point Likert scale was used, 5 meaning “strongly agree,” 4 meaning “relatively agree,” 3 meaning “generally,” 2 meaning “relatively disagree,” and 1 meaning “Strongly disagree.” To ensure the accuracy of the scale, the questionnaire was reviewed and revised by experts in the research field and individuals or organizations with entrepreneurial experience.

### Reliability Test

The statistical software used in this study was SPSS25.0. There are 13 specific indicators for the investment mechanism of EE. The results of descriptive statistics showed that the minimum value of the measurement index was 1, the maximum value was 5, and the sample individuals had certain differences. Additionally, the mean was between 3.55 and 3.80, the variance was between 0.836 and 0.947, and the standard deviation was between 0.914 and 0.973, indicating that the sample difference was small and the evaluation results had good consistency (see Table 2). The scale passed the internal consistency test, and the Cronbach Alpha value was 0.972, indicating a good reliability.

**Table 2:** Descriptive statistics of investment mechanism of entrepreneurship education (descending order of mean).

**Entrepreneurship education (*N* = 13)**	**Minimum**	**Maximum**	**Mean**	**Standard deviation**	**variance**
X12 Entrepreneurship practice has independent college students entrepreneurship parks	1	5	3.80	0.919	0.844
X4 Teachers have rich experience in teaching entrepreneurship education	1	5	3.72	0.948	0.899
X13 Entrepreneurship practice has special off-campus practice bases	1	5	3.72	0.930	0.865
X10 Entrepreneurship practice is supported by special venture funds	1	5	3.71	0.938	0.879
X11 The school provides integrated entrepreneurship practice services	1	5	3.70	0.914	0.836
X2 Teachers teach in a variety of ways	1	5	3.69	0.942	0.887
X7 There are many different kinds of entrepreneurship competitions	1	5	3.68	0.932	0.869
X6 Entrepreneurship course content and the forefront of The Times closely combined with the trend	1	5	3.66	0.926	0.858
X1 Entrepreneurship education courses vary in types	1	5	3.61	0.953	0.908
X3 Teachers have entrepreneurial experience	1	5	3.60	0.971	0.943
X9 Entrepreneurship competition projects and professional integration degree is high	1	5	3.60	0.936	0.876
X8 Entrepreneurship competition projects are easier to land	1	5	3.55	0.943	0.890
X5 Entrepreneurship course content is closely integrated with the professional knowledge you have learned	1	5	3.55	0.973	0.947

EEP is measured by 5 indicators, with the minimum mean value of 3.82 and the maximum value of 3.88. The minimum and maximum variance was 0.761 and 0.784, respectively. The minimum standard deviation was 0.872 and the maximum was 0.885 (see Table 3). The scale passed the internal consistency test, and the Cronbach Alpha value was 0.973, indicating a good reliability.

**Table 3:** Descriptive statistics of EEP (descending order of mean).

**EEP (*N* = 5)**	**Minimum**	**Maximum**	**Mean**	**Standard deviation**	**variance**
P2 Entrepreneurship education is helpful to cultivate the spirit of innovation	1	5	3.88	0.878	0.771
P3 Entrepreneurship education is helpful to improve entrepreneurial skills	1	5	3.87	0.877	0.768
P4 Entrepreneurship education is helpful to stimulate the willingness to start a business	1	5	3.87	0.872	0.761
P1 Entrepreneurship education is helpful to enrich entrepreneurial knowledge	1	5	3.86	0.876	0.767
P5 Generally satisfied with the quality of entrepreneurship education	1	5	3.82	0.885	0.784

### Validity Test

The exploratory factor analysis of the indicators of the EE investment mechanism showed that the KMO value was 0.966 (more than 0.8), the Bartlett test significance was 0.000, the degree of freedom was 78, and the approximate chi-square was 209,437.312. It showed that the scale had good validity and was suitable for factor analysis. In the analysis, according to the method of “characteristic root > 1,” only one common factor could be obtained, and the total variance explanation was only 74.656%. Therefore, combined with theoretical assumptions, the analysis was conducted by extracting the “fixed number of factors = 3.” The results showed that the total variance interpretation reached 84.744%. After matrix rotation, the variables with component factor score >0.5 were classified as a common factor, and finally three common factors were obtained, which named as “EC” (factor 1), “EP” (factor 2), and “IEEPE” (factor 3). The specific analysis results are shown in Table 4.

**Table 4:** Factor analysis results of investment mechanism of entrepreneurship education.

	**Factors**		
	**1**	**2**	**3**
X2	0.792	0.345	0.350
X4	0.777	0.340	0.337
X3	0.767	0.328	0.372
X1	0.753	0.339	0.378
X12	0.325	0.831	0.272
X13	0.328	0.797	0.363
X11	0.373	0.750	0.410
X10	0.331	0.725	0.429
X5	0.423	0.313	0.756
X8	0.379	0.399	0.728
X9	0.345	0.452	0.713
X6	0.461	0.385	0.700
X7	0.451	0.426	0.666

*Extraction method: principal component analysis.*

*Rotation method: Kaiser normalized maximum variance method.*

*^a^The rotation has converged after 6 iterations*.

Similarly, exploratory factor analysis was conducted on all indicators of EEP. The results showed that KMO value was 0.917 (more than 0.8), Bartlett test significance was 0.000, degree of freedom was 10, and approximate chi-square was 98377.352, indicating that the scale validity was good and suitable for factor analysis. According to the method of “characteristic root > 1,” a common factor was obtained, which was named as “EEP.” The total variance was interpreted as 90.267%. The analysis results are shown in Table 5.

**Table 5:** Factor analysis results of EEP.

**Variance of common factor**		
	**Initial**	**Extract**
P1	1.000	0.907
P2	1.000	0.912
P3	1.000	0.919
P4	1.000	0.910
P5	1.000	0.865

*Extraction method: principal component analysis*.

## Results

Based on the above analysis, regression analysis was conducted with “EEP” as the dependent variable; “EC,” “EP,” and “IEEPE” as the independent variables; and “entrepreneurial choice” and “household registration before college entrance examination” as the control variables. Since regression analysis requires the independent variables to be equidistant or equi-proportional, the two control variables need to be converted to dummy variables. For entrepreneurial choice, the original question is “What do you want to do after graduation?,” and the choice “starting a business” is “Yes,” while the other choices “employment,” “further study,” and “other” are merged into “No,” that is, “No” was taken as the reference group. The “household registration before college entrance examination” took “rural” as the reference group.

**Figure 2:**
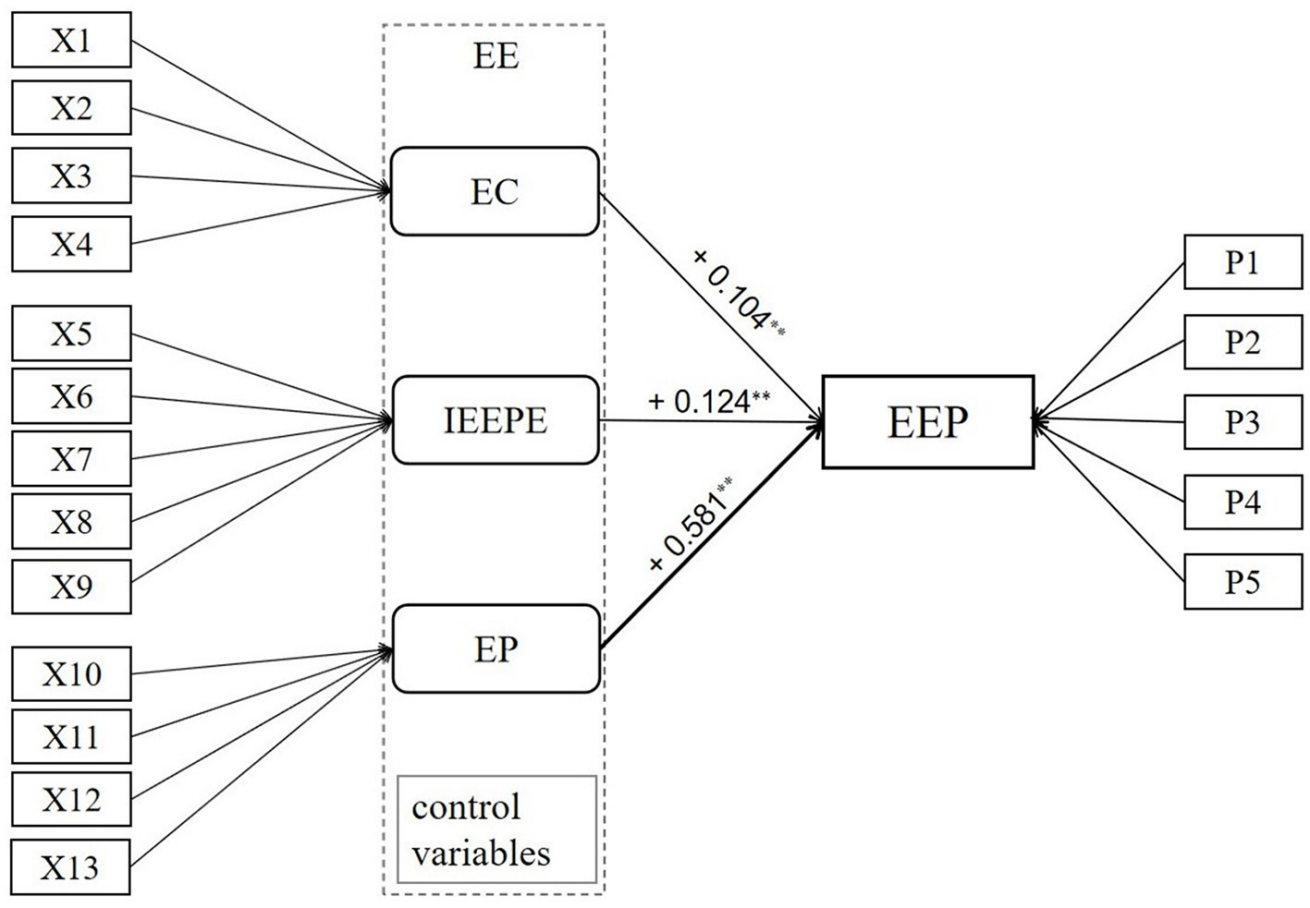
Empirical analysis of EE and EEP (whole model).

Pearson correlation analysis results showed that the respective variables were significantly correlated with the dependent variables, and the regression model passed the multicollinearity test (the minimum VIF value was 1.003 and the maximum value was 5.032). To solve the possible heteroscedasticity problem and draw a more scientific conclusion, the analysis method combining logarithm and robust standard error regression was adopted. Two steps were involved: (1) Take the logarithm of base 10 of the independent and dependent variables (control variables were not included) to generate new variables; (2) Robust standard error regression was adopted using the new variables. Since EE pays less attention to gender issues, it mainly guides women to learn the “correct” male entrepreneur mentality at present (Berggren, 2020). However, EE programs that can improve the entrepreneurial performance of men are not necessarily effective for women (Brixiova et al., 2020), and gender differences may lead to different EE needs and evaluations. Therefore, the analysis was conducted by gender; the results are shown in Table 6. The standardized regression equations of the three models are as follows, and the regression model (whole) is shown as Figure 2.

Model 1 (whole): EEP ≈ 0.104 × EC + 0.124 × IEEPE + 0.581 × EP + control variables
Model 2 (male): EEP ≈ 0.096 × EC + 0.152 × IEEPE + 0.568 × EP + control variables
Model 2 (female): EEP ≈ 0.112 × EC + 0.097 × IEEPE + 0.587 × EP + control variables.

**Table 6:** Regression model analysis results.

	**Regression coefficient**		
	**Model 1 (whole)**	**Model 2 (male)**	**Model 2 (female)**
Constant	0.127^**^ (21.261)	0.120^**^ (14.862)	0.135^**^ (15.478)
Entrepreneurial choice(Yes and No)	0.002 (1.952)	0.005^**^ (2.748)	0.001 (0.728)
household registration before college entrance examination(Urban and Rural)	−0.004^**^ (−3.477)	−0.004^**^ (−2.603)	−0.003 (−1.899)
Log10_EC	0.104^**^ (7.525)	0.096^**^ (4.358)	0.112^**^ (6.884)
Log10_IEEPE	0.124^**^ (6.998)	0.152^**^ (5.049)	0.097^**^ (5.212)
Log10_EP	0.581^**^ (32.832)	0.568^**^ (20.283)	0.587^**^ (27.461)
Sample size	13885	7008	6877
*R* ^2^	0.694	0.721	0.657
*R*^2^ after adjustment	0.694	0.720	0.656
*F*	*F* (5,13879) = 1269.096, *p =* 0.000	*F* (5,7002) = 720.488,*p* = 0.000	*F* (5,6871) = 567.834, *p =* 0.000
DW	1.943	1.946	1.928

*The dependent variable: Log10_EEP.*

*p < 0.05

** *p < 0.01 The parentheses are the values of t*.

### EP Is the Most Important Factor Affecting EEP of Higher Vocational Colleges

As can be seen from Table 6, in Model 1, EP has a significant positive impact on EEP, and the regression coefficient of EP (0.581) is the largest, followed by IEEPE and EC. In other words, EP is the most important factor affecting EEP. EE in China emphasizes practice rather than theory. This is due to the narrow understanding of EE and the explicit evaluation indicators (which make it easier to evaluate) (Huang and Lv, 2018). Compared with ordinary higher vocational colleges, “Double High-level Plan” colleges have more resources and more advantages. However, limited to education ideas, they show strong practice, weak theory, and the tendency of superficiality and formalization. Certainly, real learning environments and practice-oriented courses are conducive to the development of entrepreneurial cognition and behavior (Heinrichs, 2016; Huang et al., 2021). Piperopoulos and Dimov (2015) investigated 114 undergraduate and graduate students from four elective courses in a British university in 2010–2011 and found that practice-oriented courses gave students more self-efficacy, entrepreneurial enthusiasm, and entrepreneurial skills than theory-oriented courses. This is consistent with the students' self-perception. More than half of the students (52.1%) believed that EP could best improve their entrepreneurial ability. Further, when asked “which EP activity is more helpful?” (Multiple choice), “practice in the entrepreneurship park on campus” (60.7%) was the most frequent response, followed by “entrepreneurship competition” (45.5%), “internship in enterprise management position” (43.0%), “entrepreneurship simulation training camp” (34.8%), and “start a company off campus” (34.6%) (see Table 7). Although “entrepreneurship competition” ranks second, it tops “first important.” Nowadays, the entrepreneurship competition is recognized and welcomed by students in higher vocational colleges, and it is more popular than practice in the entrepreneurship park on campus. This conclusion can also be strengthened by the fact that 59.8% choose “entrepreneurship competition” as the first important option and “practice in the entrepreneurship park on campus” as the second important option (the reverse is only 12.4%). However, in terms of frequency, entrepreneurship competition appears to be less common. In addition, the proportion that answered “no” as the first important choice reached 13.9%, indicating the absence or poor effect of EP.

**Table 7:** Which EP activity is more helpful?

	**First important (%)**	**Second important (%)**	**Third important (%)**	**Frequency (%)**
Entrepreneurship competition	35.4	5.0	5.1	45.5
Practice in the entrepreneurship park on campus	27.2	26.7	6.7	60.6
Start a company off campus	7.4	13.5	13.7	34.6
Internship in enterprise management position	9.3	16.8	16.9	43.0
Entrepreneurship simulation boot camp	6.8	9.3	18.7	34.8
None	13.9	0.4	0.5	14.8

Teachers' guidance also plays a prominent role in EP. Students believe that it is helpful to improve professional knowledge and application ability (M = 3.90, SD = 0.864), understand the cutting-edge trends of subject knowledge (M = 3.89, SD = 0.869), improve scientific research ability (M = 3.87, SD = 0.879), improve the ability of innovation and entrepreneurship (M = 3.90, SD = 0.877), and promote the implementation of entrepreneurship projects (M = 3.85, SD = 0.885). However, most of the EE teachers in China are transferred from student work or they have economic and management background. Their entrepreneurial ability is generally weak, they are few in number, and most of them are part-time teachers (Huang et al., 2017). This is also the main reason why 46.6% of students answered “no teacher participated” when asked “major barriers to working with teachers on entrepreneurship projects.” Therefore, during EE implementation, professional human resource management strategies need to be generated to strengthening the quality of teachers (Huang et al., 2020a).

In terms of guarantee of entrepreneurship policy (multiple choice), the frequency of entrepreneurship scholarship (77.3%) is the highest, indicating that this policy is more common, followed by interest-free loan (57%), admission to entrepreneurship park (55.1%), and credit mutual recognition (16.8%). Obviously, the policy guarantee is insufficient, especially the lack of credit mutual recognition policy, due to which students are unable to resolve the contradiction between business and course learning. They cannot start a business easily because time support is highly important (Wang and Huang, 2020).

### EC and IEEPE Have Significant Positive Influence on EEP of Higher Vocational Colleges

In Model 1, both EC and IEEPE had significant positive effects on EEP, but the regression coefficients were relatively small-−0.104 and 0.124, respectively. As the most widely accepted EE method for students, EC is directly related to the quality of talent cultivation. However, the basic EC is still not fully popularized at present. Only 83.6% students think that the school offers EC, and the proportion of students who have not taken the “foundation of entrepreneurship” is as high as 30%.

In terms of teaching methods (multiple choice), “classroom teaching” is regarded as the most important and effective by most students (see Table 8), which reflects the current situation that EC in colleges is still mainly taught through theory and supplemented in other ways. Although “simulations” and “case teaching” are rated as inferior to “classroom teaching,” their total frequency is 13% and 5.3% higher, respectively. Cross analysis showed for those who chose “simulations” as the most important option, the second most important option was “case teaching” (30.6%), “group discussion” (16.6%), and “classroom teaching” (13.4%); for those who chose “case teaching” as the first important option, the second most important option was “simulations” (42.6%), “group discussion” (33.4%), and “classroom teaching” (10.4%). It can be inferred that in the context of diversified teaching methods, personalized, interactive, and experiential teaching methods are more effective than traditional teaching modes (Wilson, 2008). Additionally, it should be noted that the effectiveness of “special lecture” and “network course” ranked at the bottom, indicating that they played a small role, and their content construction and course management needs to be strengthened.

**Table 8:** Effective teaching methods for EC.

	**First important (%)**	**Second important (%)**	**Third important (%)**	**Frequency (%)**
Classroom teaching	42.8	6.5	7.0	56.3
Simulations	23.8	20.2	25.3	69.3
Case teaching	20.4	30.7	10.5	61.6
Group discussion	7.9	19.5	18.9	46.3
Special lecture	2.2	4.1	10.2	16.5
Network course	1.4	3.1	4.9	9.4
Other	1.5	0.2	0.5	2.2

As for the assessment methods of EC (multiple choice), it can be seen from Table 9 that the three assessment methods of “entrepreneurship simulation,” “showing entrepreneurship projects,” and “writing business plan” are relatively popular. “Winning prizes in entrepreneurship competitions” and “founding a company” may not be widely recognized due to their high requirements and them being applicable to a small number of people. The low evaluation of traditional and universal “theory exam” indicates that this method is not effective or satisfactory.

**Table 9:** Effective assessment methods for EC.

	**First important (%)**	**Second important (%)**	**Third important (%)**	**Frequency (%)**
Entrepreneurship simulation	26.7	24.7	20.5	71.9
Writing business plans	23.9	16.9	9.9	50.7
Theory exam	24.0	2.2	3.3	29.5
Showing entrepreneurship projects	18.0	27.5	18.5	64.0
Winning prizes in entrepreneurship competitions	3.3	9.3	14.1	26.7
Founding a company	2.5	3.1	8.4	14.0
Other	1.6	0.1	0.4	2.1

The deep IEEPE is conducive to the construction of a professional brand, thus promoting the high-quality development of colleges and fostering entrepreneurial colleges. However, IEEPE exists many problems such as low degree of integration, low level and low efficiency, and disregard of the differences among people (Peng and Zhu, 2021). Students are not satisfied with IEEPE (Huang et al., 2020b), they believe that EC is not closely related to the forefront of The Times (46.7%: refers to the total proportion of “strongly disagree,” “relatively disagree,” and “generally,” the same as below) and the subject major they learn (52.5%). There are not enough kinds of entrepreneurship competitions (45.8%) and they are not well combined with majors (50.6%). Furthermore, more than half (53.5%) students believe that entrepreneurship competition projects cannot be successfully implemented. Although colleges have launched EE under the vigorous promotion of the government, not all have taken it as a strategic priority. The EE implementation departments are often independent or affiliated to some college or leading group system. As a result, they are siloed from the implementation departments of professional education, making it difficult to promote IEEPE. This is mainly reflected in the following facts. First, professional education emphasizes professional knowledge, which is not closely related to the frontier trend and cannot keep up with the requirements of EE. Second, the advantages of professional education teachers are obvious. On average, each “Double High-level Plan” higher vocational college has 411 part-time teachers (*N* = 26), and the proportion of “double-qualified” full-time teachers is 81.94% (*N* = 40)^1^ However, these teachers from the industry have not been fully utilized; they are less involved in EE and guidance. If professional education and EE continue to operate independently, resources and information will form “isolated islands,” which will not be effectively used or may even lead to repeated input. Therefore, it is necessary to strengthen the top-level design and promote IEEPE.

### Ascribed Factors Influence Students' Evaluation of Investment in Entrepreneurship Education

As can be seen from Table 2, students in “Double High-level Plan” tend to have a general evaluation of investment in EE, with the highest score of 3.80 and the minimum score of 3.55 in the 13 indicators, showing that most students believe that investment in EE in all aspects needs to be improved. The evaluation by students is affected by many factors. Ascribed factors are inherent to individuals, including gender, family background, and other unchangeable factors. From this perspective, the independent sample *T*-test of the three dimensions of EE was further conducted by using SPSS25.0 software, and the following results were found:

1) There are significant differences in the evaluation by students of different genders; female students' evaluation of each dimension is lower than male students. For EC, female (M = 3.60, SD = 0.821) < male (M = 3.71, SD = 0.928), *p* = 0.000; for EP, female (M = 3.72, SD = 0.801) < male (M = 3.75, SD = 0.905), *p* = 0.036; for IEEPE, female (M = 3.56, SD = 0.803) < male (M = 3.65, SD = 0.912), *p* = 0.000. This may be owing to their own development orientation. After graduation, female students mainly focus on employment and further study, and the proportion (12.1%) who choose to start their own business is less than half of male students (25.4%), which may lead to female students paying less attention to EE. For example, the proportion of female students (14.3%) participating in EP is 11.2% lower than that of male students (25.5%), and the proportion of female students who have taken 3 or more ECs (8.40%) is 5.5% lower than that of male students (13.9%), which ultimately affects female students' perception and evaluation of EE investment. In addition, there are differences between male and female students in their demands for EE. Compared to female students, the demand for IEEPE is higher and the demand for EP and EC is lower among male students (see Table 6).
2) The evaluation by students whose parents have entrepreneurial experience is significantly higher than that of students whose parents have no entrepreneurial experience (*p* = 0.000). For EC, the former (M = 3.76, SD = 0.910) > the latter (M = 3.62, SD = 0.864); for EP, the former (M = 3.84, SD = 0.866) > the latter (M = 3.69, SD = 0.848); for IEEFE, the former (M = 3.72, SD = 0.889) > the latter (M = 3.57, SD = 0.848). That may be because their families (M = 3.22, SD = 1.013) have more entrepreneurial resources than other families (M = 2.49, SD = 1.052) (*p* = 0.000). EE can help them better understand business and start their own businesses.
3) Students with “only child” status and students from urban regions evaluated investment in EE much more positively than students who had siblings or hailed from rural areas (*p* = 0.000). Details are as follows: for EC, urban students (M = 3.72, SD = 0.910) > rural students (M = 3.64, SD = 0.866); for EP, urban students (M = 3.79, SD = 0.890) > rural students (M = 3.71, SD = 0.842); for IEEPE, urban students (M = 3.68, SD = 0.899) > rural students (M = 3.58, SD = 0.846). Similarly, the former two types of students have more family support, helpful social network, and other entrepreneurial resources.

All of the above is consistent with Zhu and He (2020), who surveyed 28,232 vocational college students in 31 provinces. They found that gender, household registration, whether parents are engaged in business, and other ascribed factors significantly affect students' evaluation of EE.

### Self-Achieved Factors Influence Students' Evaluation of Investment in Entrepreneurship Education

Self-achieved factors refer to the conditions, qualifications, and abilities that individuals can obtain through their own efforts. Factors such as school type and subject background can affect students' satisfaction with EE (Guo and Luo, 2020). Different types of “Double High-level Plan” higher vocational colleges have significant differences in the evaluation of investment in EE. The result of *T*-test was, the evaluation of high-level school construction colleges was higher than that of high-level major group construction colleges (*p* = 0.000). For EC, the former (M = 3.72, SD = 0.873) > the latter (M = 3.63, SD = 0.879); for EP, the former (M = 3.82, SD = 0.853) > the latter (M = 3.70, SD = 0.854); for IEEPE, the former (M = 3.68, SD = 0.862) > the latter (M = 3.58, SD = 0.859). That may be related to the entry threshold, where high-level school construction colleges are more demanding.

From the school area, analysis of variance was used, and Tamhane test was conducted due to heterogeneity of variance. The results showed that the evaluation of higher vocational students in the eastern China was significantly higher than that of central and western China: (1) the mean difference of EC between Eastern and Central, Western were 0.059 (*p* = 0.042) and 0.104 (*p* = 0.000); (2) the EP's were 0.175 and 0.195 (*p* = 0.000); and (3) similarly, the IEEPE's were 0.117 and 0.154 (*p* = 0.000). There was no significant difference between central and western China. This finding is related to the high level of economic development in the east. A strong economic foundation can promote investment in education, as reflected in the ranking of the number of “Double High-level Plan” colleges. The top four provinces, Jiangsu (20), Zhejiang (15), Shandong (15), and Guangdong (14), are all big economically developed provinces, which in turn indicates that EE investment in central and western China is relatively weak. However, further analysis showed that students in central and western China (20.72%) were significantly more likely to start their own businesses after graduation than those in eastern China (18.43%). This is because students in central and western China are more likely to have trouble finding jobs (due to low economic development in their region) and turn to self-employment (Caggiano et al., 2016). Thus, they have a great need for high-quality EE.

In terms of majors, multiple comparisons (Tamhane) results showed that economics and management students had significantly lower evaluations of EC (mean difference was 0.071, *p* = 0.000) and IEEPE (mean difference was 0.040, *p* = 0.033) than science and technology students, and significantly lower evaluations of EP (mean difference was 0.070, *p* = 0.030) than students with other majors. The science and technology as highly innovative disciplines, American universities often set up entrepreneurship centers based on their disciplinary advantages, such as the Stanford Technology Ventures Program (STVP) of Stanford Engineering. IEEPE of science and technology is better, and a technology-driven course can greatly help students develop entrepreneurial thinking (Jena, 2020), so it is obvious that those students gave better evaluations. Meanwhile, the proportion of economics and management students choosing to start their own businesses is higher, and they have higher requirements of EP, so their evaluation is not as positive as that of students with other majors.

Additionally, *T*-test results showed that students who had received social EE valued their investment in EE significantly higher than those who had not received social EE (*p* = 0.000). However, there were few courses on social entrepreneurship, and 24.3% of students said they had not taken any courses on it.

## Conclusion

Based on our distribution of 13,885 student questionnaires, our study provides valuable evidence that the three dimensions of EE have a significant impact on the EEP, with EP being the most important factor. IEEPE and EC followed with a slight gender difference. Furthermore, ascribed factors and self-achieved factors were found to affect students' evaluation of EE. Compared with the research of EE evaluation based on “process-result” by Zhu and He (2020), this study explored the impact of EE on EEP from different dimensions (such as IEEPE), and explored by gender. Currently, many scholars are exploring how to improve EEP, the paper empirically tested the effectiveness of three dimensions of EE and proved that EE is beneficial to improve college students' entrepreneurial ability and willingness, etc. This has certain theoretical and practical significance for the improvement of entrepreneurship education in other developing countries.

## Discussion

### Theoretical Implications

The findings of the study have three main theoretical implications. Firstly, this study introduces the EE in “Double High-level Plan” higher vocational colleges with Chinese characteristics which were implemented just since December 2019. Secondly, this study builds sustainable development model of EE from the perspective of students. Thirdly, this study provides detailed evidence for positive effects of EE on EEP, and differences between different student groups. These should help the academic community to better understand EE.

### Managerial Implications

The study also has some important practical implications. According to the 2020 Annual Report of Quality of Vocational Education in China, on average, only 2.02% graduates (class 2019) in each higher vocational colleges started their own businesses. These findings should help colleges to develop EE to improve entrepreneurship rate and educational quality. Combined with the previous analysis, four suggestions are formulated as follow.

Firstly, integrated development of “Double High-level” construction and EE. The overall goal of “Double High-level Plan” is to build “locally inseparable, recognized by the industry, and internationally communicable” high-level vocational colleges and ultimately serve the national strategy and economic and social development. The “double high-level” construction is closely related to EE. Combined with the above analysis, these colleges can make efforts in the following two aspects. First, they can deepen the integration of industry and education—the soul of vocational education and the key of EE. They can establish a dynamic adjustment mechanism for majors, set up cutting-edge majors, and digitize traditional majors according to industry needs. The colleges and enterprises should establish a sense of community of common destiny, make talent training plans through in-depth cooperation, and cultivate high-quality technical talents with digital operation ability and digital professional accomplishments. Second, colleges ought to strengthen IEEPE. The core of the “double high-level” construction is the building of high-level major groups. Therefore, colleges should integrate EE into major group construction so as to cultivate innovative and entrepreneurial talents in a ubiquitous environment.

Secondly, put theoretical education and practical education together. EC is important although its influence on EEP is not as great as that of EP. Only when both EC and EP are promoted simultaneously can EE be suitable for different educational goals and student groups. First of all, the educational objectives and development orientation of colleges should be clearly defined, and then the EE objectives should be further determined in combination with the advantages of colleges so as to strengthen the top-level design and make a good plan for EE. Second, a hierarchical and classified EE system should be constructed so that theoretical education covers all students, practical education benefits students interested in entrepreneurship, and elite education targets students who choose to start a business. This will help form a new EE pattern in which all people have received entrepreneurial edification, most people have experienced entrepreneurial activities, and a few people have started entrepreneurial plans. Finally, the teaching situations applicable to the two need to be correctly differentiated, and then different forms of activities should be combined into “advanced” EE according to the degree of difficulty.

Thirdly, take the digital reform of “Three Teaches” as a whole. Teachers are the key factor in the reform of “Three Teaches” (teachers, teaching materials, and teaching methods). At present, there is a serious shortage of EE teachers, and the teachers' educational ability is generally weak. Moreover, teaching materials and methods are lagging behind. Therefore, it is urgent to use information technology to promote the overall improvement of EE quality in a better and faster way. The first measure is to improve teachers' digital literacy. It is important to establish and improve the training and practice system. And high-level, structured, and wide-ranging teaching innovation teams also should be set up to help teachers improve the “double-qualified” quality and cultivate EE ability. Teachers need to be encouraged to extensively participate in EE and guidance. The second measure is to speed up the informatization of teaching materials. Frontline teachers need to be encouraged to compile effective ECs that students like into teaching materials. Colleges and enterprises should act as “double editor-in-chief” to jointly develop new loose-leaf and work manual-style textbooks that are based on school and regional characteristics, and suitable for different students. Further, a high-quality video course and network course resource database should be created to enable anytime, anywhere learning. The last measure is to promote the diversified development of teaching methods. Teachers need to be encouraged to teach in accordance with students' aptitude, and implement heuristic, discussion-based, and experiential teaching methods. Big data, cloud computing, AI, VR/AR, and other modern educational technologies should be introduced to create digital teaching mode.

Finally, develop EE in a comprehensively and balanced way. First of all, macroscopically, correct the situation of imbalanced development of EE. Do this by formulating east-west cooperation plans, using digital platforms to promote inter-regional exchanges, mutual learning and resource sharing, and formulating preferential policies to promote EE in central and western colleges. Second, at the middle level, strengthen the investment in EE and encourage these colleges to be EE “leaders” to boost the overall EE quality of more than 1,400 colleges in China. Finally, microscopically, grasp the needs of student groups of different genders, subject majors, and backgrounds to provide EE in a targeted way. For example, introduce female role models to promote EE among female students (Berggren, 2020). Encourage interdisciplinary cooperation to achieve complementarity among disciplines. Encourage government, enterprise, industry, and colleges work together to establish and improve environmental support systems such as zero-interest loans, site leasing, entrepreneurship guidance, and credit mutual recognition.

### Limitations and Further Research Opportunities

The limitation of this study is that it only evaluates from the perspective of students. Asking students to directly evaluate through questionnaires or indirectly evaluate factors composed of multiple indicators may lead to reporting bias, especially for structures that are difficult to define or identify (Purzer et al., 2016). The research on this aspect can be further strengthened through the investigation of schools and teachers.

## Data Availability Statement

The original contributions presented in the study are included in the article, further inquiries can be directed to the corresponding authors.

## Ethics Statement

Ethical approval was not required in accordance with laws, regulations, and institutional requirements. Completion of the survey implied the participants' informed consent.

## Author Contributions

YH: funding acquisition, project administration, supervision, and writing-review. YZ: conceptualization, methodology, writing-original draft, and editing. ZL and DX: questionnaire survey. RZ: questionnaire survey and editing. All authors contributed equally to the article and approved the submitted version.

## Funding

The phased results of the key project of the National Social Science Fund, Research on Barriers and Policy Support Mechanisms for Female Entrepreneurship in the Digital Era (20ASH012).

## Conflict of Interest

The authors declare that the research was conducted in the absence of any commercial or financial relationships that could be construed as a potential conflict of interest.

## Publisher's Note

All claims expressed in this article are solely those of the authors and do not necessarily represent those of their affiliated organizations, or those of the publisher, the editors and the reviewers. Any product that may be evaluated in this article, or claim that may be made by its manufacturer, is not guaranteed or endorsed by the publisher.

## Footnotes

^1^The number of part-time teachers and the proportion of “double-qualified” teachers are calculated from the 2019 quality annual report of 40 sample colleges published by Higher Technical and Vocational Education in China. https://www.tech.net.cn/column_rcpy/index.aspx.
